# The Pharmacological Mechanisms of Xiaochaihutang in Treating Breast Cancer Based on Network Pharmacology

**DOI:** 10.1155/2022/3900636

**Published:** 2022-03-09

**Authors:** Lin Zheng, Hongnan Jiang, Ruoqi Li, Liying Song, Ruihan Chen, Honglin Dong

**Affiliations:** ^1^Department of Vascular Surgery, The Second Hospital of Shanxi Medical University, Taiyuan 030000, China; ^2^Department of Breast Surgery, The Second Hospital of Shanxi Medical University, Taiyuan 030000, China; ^3^General Surgery Department, Third Hospital of Shanxi Medical University, Shanxi Bethune Hospital, Shanxi Academy of Medical Sciences, Tongji Shanxi Hospital, Taiyuan, 030032, China; ^4^Shanxi Medical University, Taiyuan, Shanxi 030607, China; ^5^First Clinical Medical College, Shanxi Medical University, Taiyuan 030001, China

## Abstract

**Background:**

As a classic prescription in Chinese medicine treatment, Xiaochaihutang (XCHT) can improve the clinical effect and reduce serum tumor markers in patients with breast cancer (BC). However, there has not been any study to confirm the mechanism. We used bioinformatics analysis and network pharmacology to find the potential targets.

**Methods:**

The differentially expressed genes (DEGs) of BC were identified from the Cancer Genome Atlas (TCGA) dataset. Then, we utilized weighted coexpression network analysis (WGCNA) with the same dataset. The target genes of BC were obtained by comparing genes of DEGs and in significant modules of WGCNA. Drug targets of XCHT from the Traditional Chinese Medicine Systems Pharmacology Database and Analysis Platform (TCMSP) database were intersected with the targets of BC. The protein-protein interaction (PPI) of the drug targets was analysed by using the STRING database. We utilized the Gene Ontology (GO) and Kyoto Encyclopedia of Genes and Genomes analysis (KEGG) enrichment analysis to identify the specific pathways and key target proteins. Receiver operator characteristic (ROC) curve was used as the verification of drug targets. Molecular docking was performed to visualize the patterns of interactions between the effective molecule and targeted protein.

**Results:**

We obtained a set of 21 target genes, which mainly encode neurotransmitter receptors or related transporters, such as OPRD1, 5-HT2A, and so on. In addition, enrichment analyses of 21 target genes showed that they were mainly concentrated in pathways related to the nervous system. Molecular docking was performed on the target gene of BC. Six active compounds can enter the active pocket of target gene, namely, naringenin, beta-sitosterol, coumestrol, nuciferine, beta-sitosterol, and protopine, thereby exerting potential therapeutic effects in BC.

**Conclusions:**

Our analysis shows that the mechanism of XCHT in the treatment of BC is mainly acting on the neurogenesis in the microenvironment of breast tumor tissue.

## 1. Background

Breast cancer (BC) is one of the most common malignant cancers in women, which will seriously affect the quality of patients' daily life. The early clinical symptoms of the disease are not obvious, and it is difficult to find smaller lesions in examinations. Therefore, early diagnosis is prone to misdiagnosis and miss. Once more obvious clinical symptoms occur, most of them need to implement radical mastectomy or even miss the best time for surgery [1]. For BC patients who are at an advanced stage and cannot be treated with surgery, adjuvant chemotherapy is indicated to reduce the grade of the tumor, decrease the blood supply to the tumor, shrink the size of the tumor, and increase the chance of surgical treatment. [2]. Traditional Chinese medicine believes that chronic conditioning combined with chemotherapy has a certain clinical value. Zhongquan divided patients with advanced BC into a control group without Xiaochaihutang (XCHT) and an experimental group with XCHT and observed a decrease in the production of tumor markers [3]. In the process of diagnosis and treatment, Baoyi found that XCHT could effectively improve the clinical symptoms of patients [4]. However, the current research on the effect of XCHT in treating BC is still in the clinical observation stage and the mechanism is unclear.

As a new method, the genome-wide molecular atlas has been used to study the occurrence of cancers. It has been widely proven to be an efficient research method. We tried to find key genes and crucial pathways for BC progression by analysing gene expression profiles and clinical results of public datasets. The academic term of network pharmacology was proposed in 2007 [5]. It is a network of ideas based on diseases, genes, and target drugs, revealing the potential mechanism of drugs acting on people. In recent years, more and more traditional Chinese medicines have been obtained through network pharmacology and get a better understanding. XCHT, as traditional Chinese medicine, has been studied in the treatment of pneumonia and other diseases [6]. Molecular docking refers to the process that a small molecular is spatially docked into a macromolecular and can score the complementary value at the binding sites, which is used for structure-based drug design. In this study, we explored the molecular mechanism of the action of XCHT in BC using network pharmacology and molecular docking. XCHT is often used to treat advanced BC with chemotherapy in the clinic, but its mechanism in the treatment of breast adenocarcinoma has not been reported. We combine differential gene analysis in bioinformatics, WGCNA, and network pharmacology to analyse targets of XCHT on BC.

## 2. Materials and Methods

### 2.1. Microarray Dataset of Breast Adenocarcinoma

A gene expression profiles of breast adenocarcinoma were downloaded from the Cancer Genome Atlas (TCGA) database (http://cancergenome.nih.gov/), which includes 139 noncancerous samples and 1108 cancerous tissues samples.

### 2.2. Identification of Differentially Expressed Genes (DEGs)

To obtain DEGs between normal and breast adenocarcinoma samples, we set |log_2_FC (fold change)| ≥1 and *p* value <0.05. An advanced volcano plot was performed using the OmicStudio tools at https://www.omicstudio.cn/tool.

### 2.3. Weighted Coexpression Network Analysis (WGCNA)

The TCGA dataset was used to construct the WGCNA network by the WGCNA R package (https://cran.rproject.org/web/packages/WGCNA/index.html) to identify genes related to the condition of breast adenocarcinoma patients. The soft threshold power of *β* was 6. We used the WGCNA package to carry out a correlation study on the clinical factors of breast adenocarcinoma patients.

### 2.4. Prediction of XCHT Targets

The XCHT formulas were Radix Bupleuri, Scutellariae Radix, *Panax ginseng* C. A. Mey., *Arum ternatum* Thunb., licorice, *Zingiber officinale* Roscoe, and Jujubae Fructus. The chemical constituents in compounds were collected on the Traditional Chinese Medicine Systems Pharmacology Database and Analysis Platform (TCMSP) (http://tcmspw.com/tcmsp.php). We set oral bioavailability (OB) ≥30%, and drug likeness (DL) was ≥0.18. OB reflects the absorption rate of oral drugs into the circulation through the liver after being absorbed by the gastrointestinal tract. DL refers to the structural similarity between herbal ingredients and known drugs.

### 2.5. Screening Targets Interacting with the Active Components of XCHT

Through intersecting by DEGs genes, clinical genes by WGCNA, and genes related to the active XCHT components, a Venn diagram (https://www.omicstudio.cn/tool) was created to obtain target genes.

### 2.6. Establishment of the Protein-Protein Interaction (PPI) Network

First, we uploaded the intersection genes onto STRING 11.0 (http://string-db.org/cgi/input.pl) to obtain the relationship of PPI. Then, the correlation and the network of targets for drugs and active ingredients were obtained by Cytoscape v3.7.2.

### 2.7. Enrichment Analysis of the Potential Target Genes

We analysed the potential target genes and their roles in signal pathways and discussed their functions. We used a feature enrichment and annotation tool based on Gene Ontology (GO) and Kyoto Encyclopedia of Genes and Genomes (KEGG). Data were obtained by the clusterProfiler R packages.

### 2.8. Verification of Target Genes and Molecular Docking Technology

The relevant dataset was searched in the TCGA, and receiver operator characteristic (ROC) curve was created as a verification of the key target of XCHT in OmicStudio tools. Subsequently, the 2D structure for the molecule ligands (naringenin, beta-sitosterol, coumestrol, nuciferine, and protopine) was downloaded from the PubChem database (https://pubchem.ncbi.nlm.nih.gov/). Use the RCSB PDB database (https://www.rcsb.org/) to retrieve and download structure files for key target proteins including ADIPOR (PDB ID: 5LXG), CYP2B6 (PDB ID: 3IBD), DRD2 (PDB ID: 7JVR), HTR2A (PDB ID: 6A94), and OPRD1 (PDB ID: 4N6H). Schrodinger software was used to calculate and export the 3D structure by minimizing energy. The target protein of ADRA1A was obtained by the Swiss model. Before docking, the ligands and acceptors need to minimize energy. Schrodinger's protein preparation wizard was used to process the protein, like deleting the water molecules of acceptors (PDB files), adding polar hydrogen atoms, repairing the bond information, repairing the peptide, and finally minimizing the energy of the protein and optimization of geometric structure. All parameters are set by default except for special instructions. Finally, the SP method was used to dock the receptor protein with the small molecule ligand of XCHT active compound.

## 3. Results

### 3.1. DEGs Identification

**Figure 1:**
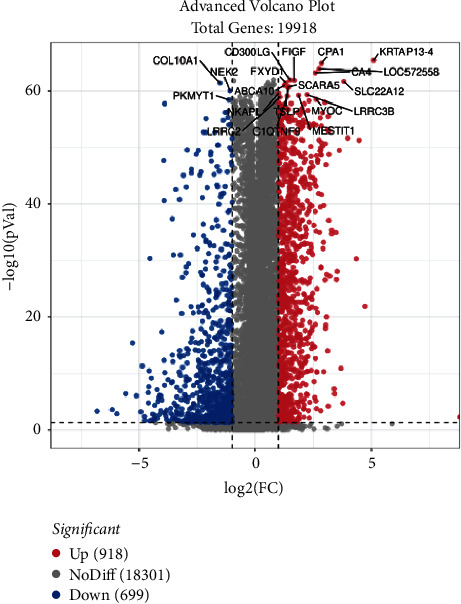
Volcano plot for DEGs. DEGs between noncancerous samples and cancerous tissues samples screened from the TCGA BC database. The red nodes are upregulated genes and the blue nods are downregulated. Identification of clinical module genes of BC.

Through secondary mining and analysis of the TCGA array database, 1617 differentially expressed genes were obtained in the noncancerous samples and cancerous tissues samples. There are upregulated 918 genes and downregulated 699 genes. Among these genes, CPA1, CA4, SLC22A12, and so on showed significant differences. The volcano map is shown in Figure 1.

### 3.2. Identification of Clinical Module Genes of BC

**Figure 2:**
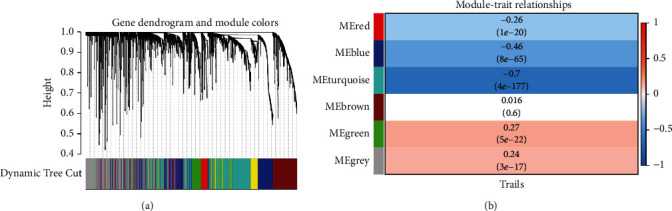
(a) Weighted gene coexpression network module in the TCGA datasets. (b) Heatmap of the correlation between modular feature genes and breast adenocarcinoma clinical features.

The TCGA dataset, in which the number of samples is 1218 and the number of genes is 20252, was used for WGCNA network analysis. Genes in the top 20% of variance were screened for analysis, and 5063 genes were obtained. We set the soft threshold to 6 (*R*^2^ = 0.85) to construct a scale-free network. Six modules were identified, including red, blue, turquoise, brown, greed, and grey modules (Figure 2(a)), and the yellow module was removed. According to the results of modules clinical relationship results (Figure 2(b)), the gene number of each module with *p* value <0.05 is given in Table 1.

### 3.3. Identification of Drug Targets of XCHT in BC

In this study, DrugBank and TCMSP databases were searched for drug targets of XCHT, the specific information of the active components of the single herb of XCHT. Target prediction of the above active components was carried out by using related target prediction techniques, and 265 targets were obtained (Supplementary Table 1).

### 3.4. Prediction of Targets of XCHT in BC and Construction of the PPI Network

**Figure 3:**
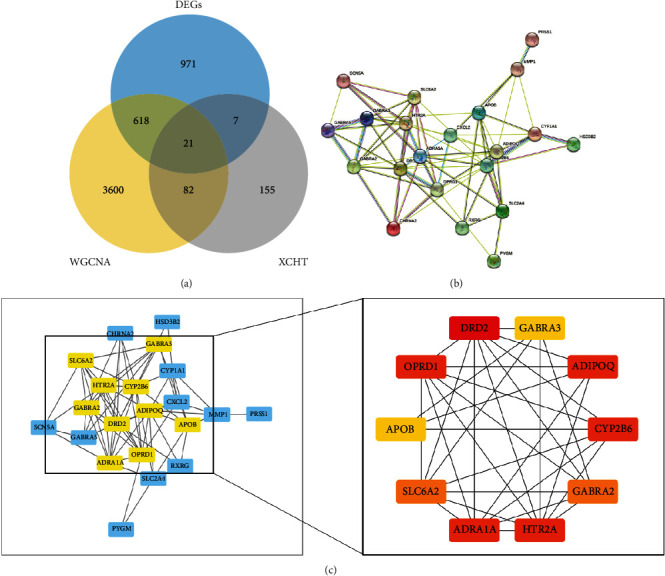
(a) The Venn diagram between the drug and the disease. It shows the overlapping of targets of breast adenocarcinoma and XCHT. (b) 21 cross-targets based on XCHT constructs a protein-protein interaction (PPI) network, which suggests how XCHT acts on BC through these targets. Each node represents different proteins and their structures, and the thickness of the wire represents the intensity of data support. (c) The PPI network of hub genes. We imported the node data downloaded from the STRING website into Cytoscape v3.7.2 software for redrawing, screened out 10 nodes with degree ≥7, and showed the relationships among these 10 nodes.

We used Venn diagrams to take the intersection of genes related to the active XCHT ingredients and breast adenocarcinoma-related genes and got 21 targets (Figure 3(a)). Using the STRING software with confidence of 0.565 indication correlation, the PPI contains 21 nodes and 69 connections (Figure 3(b)). There are 21 nodes in the PPI diagram, and each node represents a target gene. In the centre of the dot is the structure diagram of the target protein. We downloaded the “TSV” format file from the STRING website and imported it into the Cytoscape v. 3.7.2 software. Then, the CytoHubba application was used to calculate the target scale to screen the core genes and observe the closeness between them. Through the statistical analysis, the number of adjacent genes connected by each target gene was calculated, and the number of adjacent genes greater than 7 was plotted again (Figure 3(c)). We screened out 10 core targets for XCHT treatment of breast adenocarcinoma, among which the red, orange, and yellow nodes represent the degree value gradually decreasing from large to small. DRD2, CYP2B6, ADIPOQ, and OPRD1 may be the significant genes for XCHT treating in BC.

### 3.5. Construction of a Drug-Target Network

**Figure 4:**
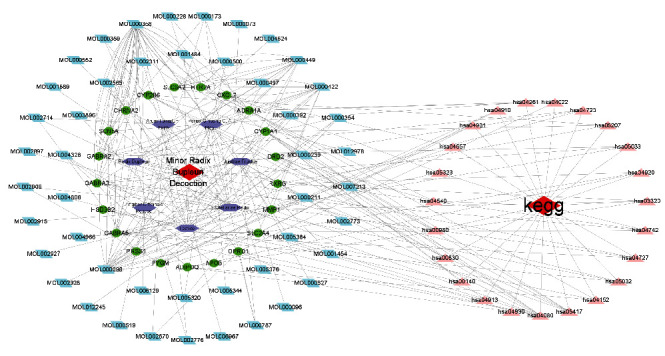
The drug-component-target network.

After the deletion of repeated genes, 265 drug targets, 21 therapeutic targets, 7 XCHT herbs, their active components, and the 21 genes enriched KEGG pathway were introduced into Cytoscape v3.7.2 to form a drug-component-target network, as shown in Figure 4. The red diamond in the big circle on the left is the Chinese medicine prescription XICH, the purple hexagon is the Chinese medicine composition of the prescription, the green circle is the 21 therapeutic targets, the blue diamond is the active ingredients in each Chinese medicine, the small circle on the right shows the KEGG pathway enriched by 21 targets, and the red diamond is KEGG. The enriched pathways are on the red triangles.

### 3.6. Analysis of Target Pathways

**Figure 5:**
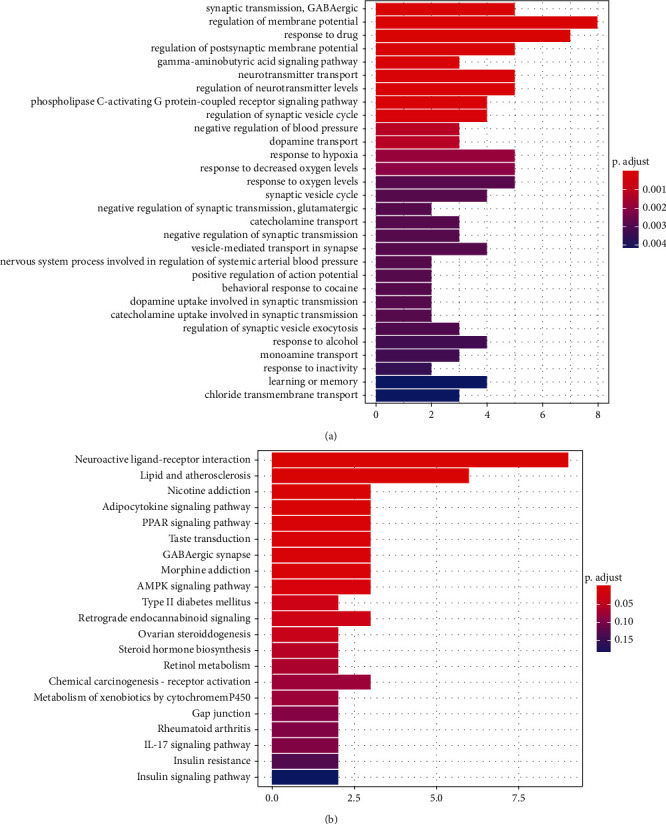
(a) Enrichment analysis of GO and BP of 21 genes about XCHT related to breast adenocarcinoma. (b) The 21 targets of the KEGG pathway and XCHT and BC.

The biological process (BP) analysis in GO enrichment analysis of 21 genes resulted in a total of 186 entries (*p* value <0.05), among which the most significant difference is synaptic transmission. Thirty of the most diverse BP analyses were presented as shown in Figure 5(a).

Twenty-one signalling pathways were obtained through KEGG enrichment analysis (*p* value <0.05), which may be used for drug therapy of BC through these 21 target genes shown in Figure 5(b). The most distinct pathways include the neuroactive ligand–receptor interaction, nicotine addiction, and adipocytokine signalling pathway.

### 3.7. Evaluation and Validation of Target Genes with ROC

**Figure 6:**
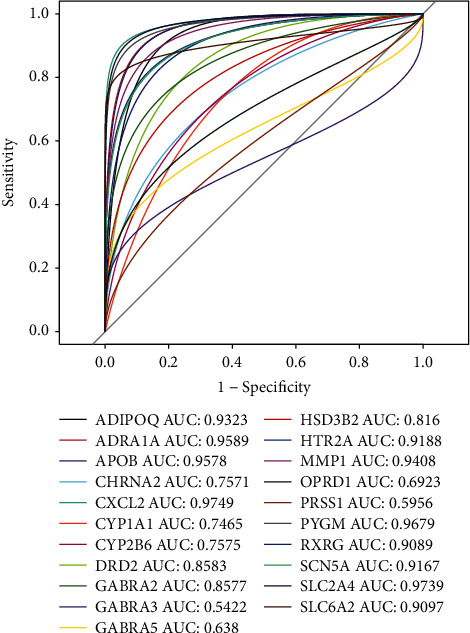
ROC curve of 21 target genes.

After obtaining data from the TCGA database, we performed ROC analysis on 21 therapeutic targets to achieve validation (Figure 6). *P* value <0.05 was considered to be a difference. AUC size is correlated with predictive power. By observing the AUC area, AUC of all target genes is >0.5, indicating that all target genes are associated with BC.

### 3.8. Results of XCHT Target Molecule Docking

**Figure 7:**
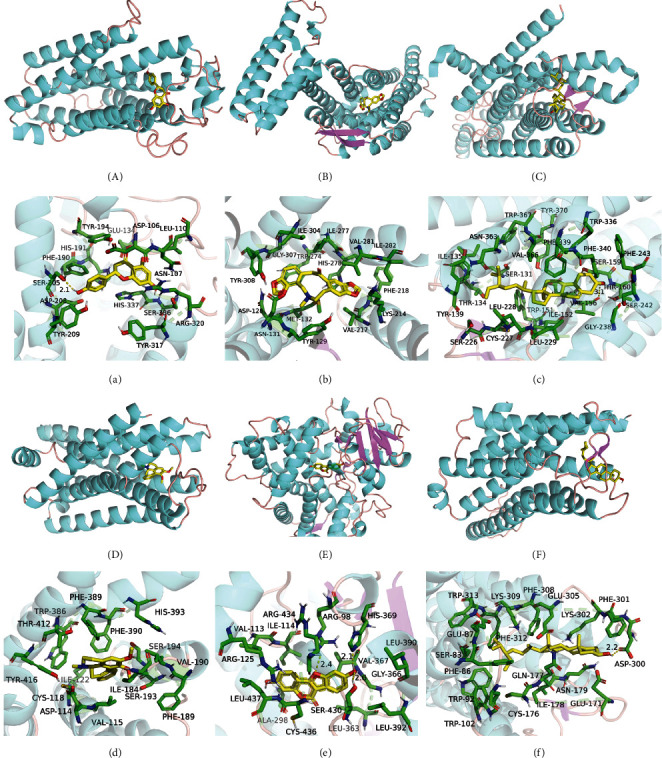
The binding mode of compound with target protein. (A–F) The 3D structure of ADIPOR, ADRA1A, CYP2B6, DRD2, HTR2A, and OPRD1 with naringenin, beta-sitosterol, coumestrol, nuciferine, beta-sitosterol, and protopine. (a–f) The detail binding mode of each compound with the active site of ADIPOR, ADRA1A, CYP2B6, DRD2, HTR2A, and OPRD1. The backbone of protein was rendered in the tube and colored in bright blue. Compounds are rendered by yellow. Yellow dash represents hydrogen bond distance.

In this study, ADIPOR, ADRA1A, CYP2B6, DRD2, HTR2A, and OPRD1 target protein were the top 6 largest degrees performing molecular docking with the core bioactive ingredients naringenin, beta-sitosterol, coumestrol, nuciferine, beta-sitosterol, and protopine. The lower the energy required for binding, the easier it is to bind to the target protein. The binding energy was less than −6 kcal/mol, which showed that the compound and the target protein had a good binding effect (Table 2). The complex formed by the docking compound and protein is shown in Figure 7 by using Pymol2.1 software. Therefore, these compounds and target protein match well and have close binding.

## 4. Discussion

BC has always been regarded as the most common malignant cancer in the world, and it is also the main cause of cancer deaths in women [7]. Although there have been significant advances in BC diagnosis and treatment methods and concepts in recent years, there are still a series of problems that need to be solved urgently, such as BC prediction targets are diverse and inconsistent; metastatic BC is prone to relapse after treatment, and prognosis is poor; BC treatment drugs are prone to drug resistance. Therefore, further search for new therapeutic targets, exploring drugs and molecular mechanisms for the treatment of BC, and the prevention of its recurrence are still the main hotspots of BC research.

Past studies of XCHT in treating BC were mostly limited to clinical observation [8–10]. The records of this prescription in traditional Chinese medical books were mostly focused on its effects on inflammation. This is also the reason why XCHT is commonly used with chemotherapy to treat advanced BC in China [11]. This study uses the TCGA database to screen target genes, the WGCNA analysis method to screen clinically relevant genes, and the TCMSP database to find the target of XCHT. After getting the intersection genes, enrichment analysis was performed to conclude the treatment mechanism. The innovative discovery of XCHT for advanced breast adenocarcinoma is that the filtered proteins are mostly neurotransmitters. Meanwhile, the pathways and functions are mostly related to the nerve infiltration of BC.

It is worth noting that all among the 21 genes screened out, 9 genes including OPRD1 (opioid receptor delta 1), 5-HT2A, DRD2, GABRA2, GABRA3, GABRA5A, CHRNA2, DRA1A, and SLC6A2 encode neurotransmitter receptors or related transporters. Many scholars have proposed that the nervous system will participate in the pathogenesis of BC in the form of axon exogenesis [12, 13]. From this, we suspect that XCHT may affect the expression of neurotransmitter-related receptor genes and then influence the nerve infiltration in the tumor microenvironment of BC, which ultimately achieves the purpose of treating BC.

OPRD1 encodes *δ*-opioid peptide receptors (DORs). High expression of DORs has been observed in tissue samples of BC patients and is found to be related to the adverse effects and poor prognosis of BC patients [14]. DRD2 is used to encode dopamine D2 receptors and will influence BC progression by different mechanisms. Matthew et al. proved that DRD2 promotes the self-renewal of triple-negative BC cells through a STAT3 and IL-6-dependent mechanism [15]. GABRA2, GABRA3, and GABRA5 are the protein codes of GABA-A receptor *α*2, *α*3, and *α*5, respectively. The two enrichment methods also enriched the GABAergic signalling pathway. Zhang et al. found that GABABR agonists can significantly promote BC invasion and migration [16]. This process is mediated by the ERK1/2 pathway, while GABABR antagonizes agents can reverse this process [17].

CYP2B6 encodes members of the cytochrome P450 superfamily [18]. Justenhoven et al. established a specific MALDI-TOF MS genotyping test and observed CYP2B6∗6 polymorphic variants (such as CYP2B6_516_T). CYP2B6_785_G increases the risk of BC [19]. According to the prediction of clinical models, we found that CYP2B6 has a good prognostic value for the prognosis of breast cancer. Meanwhile, it is easily combined with coumestrol, which is one of the core bioactive ingredients of XCHT. Therefore, reducing CYP2B6 levels is a potential strategy for BC treatment.

ADIPOQ encodes adiponectin secreted by fat cells in a breast tumor microenvironment and negatively regulates cancer cell growth. ADIPOQ/adiponectin can induce cell cycle arrest and apoptosis, increase the expression of BAD, TP53, and PTEN, decrease the expression of antiapoptosis genes BCL2 and BIRC5/survivin [20]. Furthermore, ADIPOQ/adiponectin induces autophagic cell death in BC, and STK11/LKB1-AMPK-ULK1 axis is consistently involved in ADIPOQ/adiponectin-mediated autophagy [21]. ADIPQ is one of the hub genes in the mechanism of XCHT in the treatment of BC and has a strong correlation with the result of BC. Consequently, ADIPOQ may be the target of XCHT therapy in BC.

Although it has not been reported that PRSS1 and SLC2A4 affect the progression of BC, our findings may help to explore the molecular mechanism of traditional Chinese medicine in the treatment of BC.

## 5. Conclusions

In conclusion, we predicted the possible pathway and mechanism of XCHT by screening target genes and analysing their function enrichment. Furthermore, verification was carried out by molecular docking. XCHT, a traditional Chinese medicine prescription, is associated with nerve invasion of BC, which provides a new direction for the study of therapeutic mechanisms in the future.

## Figures and Tables

**Table 1:** Screening clinical genes by WGCNA color modules.

Module	Number	*P*
Red	235	1*e* − 20
Blue	785	8*e* − 65
Turquoise	1608	4*e* − 177
Green	239	5*e* − 22
Grey	1454	3*e* − 17
Sum	4321	

**Table 2:** The selected compounds of docking results.

Target	Compounds	Structure	Docking score (kcal/mol)	Combination type
ADIPOR	Naringenin	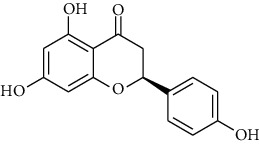	−6.91	Hydrogen bonds, hydrophobic interactive
ADRA1A	Beta-sitosterol	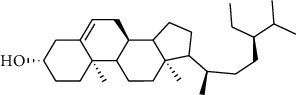	−8.08	Hydrogen bonds, hydrophobic interactive
CYP2B6	Coumestrol	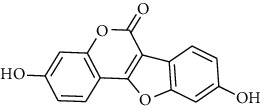	−7.44	Hydrogen bonds, hydrophobic interactive
DRD2	Nuciferine	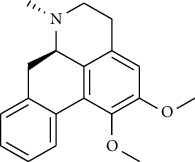	−7.92	Hydrogen bonds, hydrophobic interactive
HTR2A	Beta-sitosterol	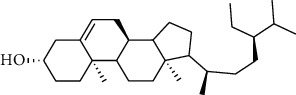	−7.52	Hydrogen bonds, hydrophobic interactive
OPRD1	Protopine	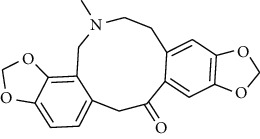	−7.15	Hydrogen bonds, hydrophobic interactive

## Data Availability

The data generated or analysed during this study are included within the article and its supplementary information files.

## Abbreviations

XCHT: Xiaochaihutang
BC: Breast cancer
DEGs: Differentially expressed genes
TCGA: The cancer genome atlas
WGCNA: Weighted coexpression network analysis
PPI: Protein-protein interaction
GO: Gene ontology
KEGG: Kyoto Encyclopedia of Genes and Genomes
ROC: Receiver operator characteristic
BP: Biological process
OPRD1: Opioid receptor delta 1
DORs: *δ*-Opioid peptide receptors
EMT: Epithelial-mesenchymal transition
TNBC: Triple-negative BC
GABA: Gamma-aminobutyric acid
GABA-A receptor: Gamma-aminobutyric acid type A receptor
CSCs: Breast cancer stem cells
NaV: Voltage-gated sodium channel
INa: Rapid and short-lived depolarizing Na+ currents
ABAT: *γ*-Aminobutyric acid transaminase
BLBC: Basal cell-like breast cancer.

## Supplementary Materials

Supplementary Materials Target prediction of the above active components was carried out by using related target prediction techniques, and 265 targets were obtained (Supplementary Table 1).
