# Supplementary material for: The Pharmacological Mechanisms of Xiaochaihutang in Treating Breast Cancer Based on Network Pharmacology
Source: Contrast Media Mol Imaging. 2022 Mar 9;2022:3900636. doi: 10.1155/2022/3900636 (PMC8926522; doi:10.1155/2022/3900636)
Supplement: Supplementary Materials — Target prediction of the above active components was carried out by using related target prediction techniques, and 265 targets were obtained (Supplementary Table 1). [file 3900636.f1.doc]

| Arum Ternatum Thunb |  |
| --- | --- |
| Mol ID | gene |
| MOL000358 | HTR2A |
| MOL002670 | HTR2C |
| MOL000449 | ALDRA |
| MOL000358 | ADRA1A |
| MOL000358 | ADRA1B |
| MOL000519 | ADRA1D |
| MOL000449 | ADRA2A |
| MOL002670 | ADRA2C |
| MOL000449 | MAOA |
| MOL000449 | MAOB |
| MOL000519 | AR |
| MOL002714 | APOD |
| MOL000358 | BAX |
| MOL000358 | BCL2 |
| MOL002714 | AHR |
| MOL000449 | ADRB1 |
| MOL000358 | ADRB2 |
| MOL000519 | DPEP1 |
| MOL000358 | PRKACA |
| MOL002670 | PDE10A |
| MOL000519 | CA2 |
| MOL000358 | CASP3 |
| MOL000358 | CASP8 |
| MOL000358 | CASP9 |
| MOL002714 | CDK1 |
| MOL000519 | CDK2 |
| MOL002714 | TP53 |
| MOL000358 | PDE3A |
| MOL000449 | CTRB1 |
| MOL002670 | F7 |
| MOL002670 | F10 |
| MOL000519 | CCNA2 |
| MOL002714 | CYCS |
| MOL000358 | DRD1 |
| MOL002670 | OPRD1 |
| MOL002714 | DPP4 |
| MOL000519 | TOP2A |
| MOL002714 | EGLN1 |
| MOL000519 | ESR1 |
| MOL002714 | FABP5 |
| MOL002714 | FOSL1 |
| MOL002714 | FOSL2 |
| MOL002714 | CCNB1 |
| MOL000358 | GABRA1 |
| MOL000358 | GABRA2 |
| MOL000358 | GABRA3 |
| MOL000358 | GABRA5 |
| MOL000358 | HSP90AA1 |
| MOL002714 | HIF1A |
| MOL000449 | IGHG1 |
| MOL002714 | IGF2 |
| MOL000449 | LTA4H |
| MOL002714 | MMP9 |
| MOL000358 | MAP2 |
| MOL000449 | NR3C2 |
| MOL000358 | OPRM1 |
| MOL000358 | CHRM1 |
| MOL000358 | CHRM2 |
| MOL000358 | CHRM3 |
| MOL000358 | CHRM4 |
| MOL002670 | CHRM5 |
| MOL002714 | MPO |
| MOL002714 | NOX5 |
| MOL000358 | CHRNA2 |
| MOL000358 | CHRNA7 |
| MOL002714 | NFATC1 |
| MOL000449 | NCOA1 |
| MOL000358 | NCOA2 |
| MOL000519 | PPARG |
| MOL000358 | PIK3CG |
| MOL000358 | KCNH2 |
| MOL000358 | PGR |
| MOL000358 | PTGS1 |
| MOL000358 | PTGS2 |
| MOL000358 | PRKCA |
| MOL002714 | FOS |
| MOL006967 | PNP |
| MOL006967 | deoD pup |
| MOL002714 | AKT1 |
| MOL000449 | RXRA |
| MOL000358 | PON1 |
| MOL000449 | SLC6A3 |
| MOL000449 | SLC6A2 |
| MOL000358 | SLC6A4 |
| MOL000358 | SCN5A |
| MOL000358 | JUN |
| MOL002714 | RELA |
| MOL000358 | TGFB1 |
| MOL002714 | PRSS1 |
| MOL002714 | TDRD7 |
| MOL002776 | PTPN1 |
| MOL000449 | PLAU |
|  |  |
| Radix Bupleuri |  |
| Mol ID | gene |
| MOL000098 | PSMD3 |
| MOL000449 | HTR2A |
| MOL000098 | MMP2 |
| MOL000098 | HSPA5 |
| MOL000098 | ACACA |
| MOL000098 | ACHE |
| MOL000098 | AHSA1 |
| MOL000422 | AKR1C3 |
| MOL000098 | ALDRA |
| MOL000449 | ADRA1A |
| MOL000422 | ADRA1B |
| MOL000449 | ADRA2A |
| MOL000449 | MAOA |
| MOL000098 | MAOB |
| MOL000098 | AR |
| MOL000422 | SLPI |
| MOL000098 | BAX |
| MOL000098 | BCL2 |
| MOL000098 | ALOX5 |
| MOL000098 | AHR |
| MOL000098 | ABCG1 |
| MOL000098 | BIRC5 |
| MOL000098 | BCL2L1 |
| MOL000449 | ADRB1 |
| MOL000098 | ADRB2 |
| MOL000098 | CRP |
| MOL000098 | CXCL10 |
| MOL000098 | CXCL11 |
| MOL000098 | CXCL2 |
| MOL000098 | PRKACA |
| MOL000098 | CASP3 |
| MOL000098 | CASP8 |
| MOL000098 | CASP9 |
| MOL000098 | CTSD |
| MOL000098 | CAV1 |
| MOL000098 | CD40LG |
| MOL000098 | CDK1 |
| MOL000354 | CDK2 |
| MOL000098 | TP53 |
| MOL000449 | CTRB1 |
| MOL000098 | CLDN4 |
| MOL000098 | F7 |
| MOL000098 | F10 |
| MOL000098 | COL3A1 |
| MOL000354 | CCNA2 |
| MOL000098 | CDKN1A |
| MOL000098 | CYP1A1 |
| MOL000098 | CYP1B1 |
| MOL000098 | CYP3A4 |
| MOL000098 | DCAF5 |
| MOL000098 | DPP4 |
| MOL000098 | TOP1 |
| MOL000098 | TOP2A |
| MOL000098 | DUOX2 |
| MOL000098 | SELE |
| MOL000098 | EGFR |
| MOL000354 | ESR1 |
| MOL000354 | ESR2 |
| MOL000098 | ELK1 |
| MOL000098 | EIF6 |
| MOL000098 | CCND1 |
| MOL000098 | CCNB1 |
| MOL000098 | GABRA1 |
| MOL000422 | GABRA2 |
| MOL000449 | GABRA3 |
| MOL004624 | GABRA6 |
| MOL000098 | GJA1 |
| MOL000354 | GRIA2 |
| MOL000098 | GSTM1 |
| MOL000098 | GSTM2 |
| MOL000354 | PYGM |
| MOL000354 | GSK3B |
| MOL000098 | HSF1 |
| MOL000098 | HSPB1 |
| MOL000098 | HSP90AA1 |
| MOL000098 | HMOX1 |
| MOL000098 | HK2 |
| MOL000098 | NKX3-1 |
| MOL000098 | HAS2 |
| MOL000098 | HIF1A |
| MOL000449 | IGHG1 |
| MOL000098 | CHUK |
| MOL000422 | IKBKB |
| MOL000098 | IGFBP3 |
| MOL000098 | IGF2 |
| MOL000098 | INSR |
| MOL000098 | ICAM1 |
| MOL000098 | IFNG |
| MOL000098 | IRF1 |
| MOL000098 | IL1A |
| MOL000098 | IL1B |
| MOL000098 | IL10 |
| MOL000098 | IL2 |
| MOL000098 | IL6 |
| MOL000098 | CXCL8 |
| MOL000098 | MMP1 |
| MOL000449 | LTA4H |
| MOL000098 | MGAM |
| MOL000098 | MMP9 |
| MOL000449 | NR3C2 |
| MOL000098 | MAPK1 |
| MOL000354 | MAPK14 |
| MOL000422 | MAPK8 |
| MOL000422 | CHRM1 |
| MOL000422 | CHRM2 |
| MOL000449 | CHRM3 |
| MOL000098 | MYC |
| MOL000098 | MPO |
| MOL000098 | NQO1 |
| MOL000449 | CHRNA7 |
| MOL000098 | NCF1 |
| MOL000098 | NFKBIA |
| MOL000098 | NOS3 |
| MOL000098 | NOS3 |
| MOL000354 | NOS2 |
| MOL000098 | NFE2L2 |
| MOL000354 | NCOA1 |
| MOL000098 | NCOA2 |
| MOL000098 | NR1I2 |
| MOL000098 | NR1I3 |
| MOL000098 | ODC1 |
| MOL000098 | SPP1 |
| MOL000354 | OLR1 |
| MOL000098 | PRXC1A |
| MOL000098 | PPARA |
| MOL000098 | PPARD |
| MOL000098 | PPARG |
| MOL000098 | PTEN |
| MOL000098 | PIK3CG |
| MOL000098 | SERPINE1 |
| MOL000098 | PARP1 |
| MOL000098 | KCNH2 |
| MOL000098 | PCOLCE |
| MOL000422 | PGR |
| MOL000098 | PTGER3 |
| MOL000098 | PTGS1 |
| MOL000098 | PTGS2 |
| MOL000098 | ACP3 |
| MOL000098 | RUNX1T1 |
| MOL000098 | PRKCA |
| MOL000098 | PRKCB |
| MOL000098 | F2 |
| MOL000098 | FOS |
| MOL000098 | NPEPPS |
| MOL000098 | AKT1 |
| MOL000098 | RAF1 |
| MOL000098 | RASSF1 |
| MOL000098 | RASA1 |
| MOL000098 | ERBB2 |
| MOL000098 | ERBB3 |
| MOL000098 | RB1 |
| MOL000098 | RXRA |
| MOL000098 | RUNX2 |
| MOL000354 | CHEK1 |
| MOL000098 | CHEK2 |
| MOL000422 | PPP3CA |
| MOL000098 | PON1 |
| MOL000098 | STAT1 |
| MOL000098 | CCL2 |
| MOL000449 | SLC6A3 |
| MOL000422 | SLC6A2 |
| MOL000098 | SCN5A |
| MOL000098 | SLC2A4 |
| MOL000098 | MMP3 |
| MOL000098 | SOD1 |
| MOL000098 | THBD |
| MOL000098 | PLAT |
| MOL000098 | F3 |
| MOL000098 | JUN |
| MOL000098 | E2F1 |
| MOL000098 | E2F2 |
| MOL000098 | RELA |
| MOL000098 | TGFB1 |
| MOL000098 | PRSS1 |
| MOL000098 | TNF |
| MOL000098 | DIO1 |
| MOL000354 | PTPN1 |
| MOL000098 | PLAU |
| MOL000098 | VCAM1 |
| MOL000098 | XDH |
|  |  |
| Scutellariae Radix |  |
| Mol ID | gene |
| MOL000358 | HTR2A |
| MOL002915 | ACHE |
| MOL000173 | AHSA1 |
| MOL000449 | ALDRA |
| MOL000228 | ADRA1A |
| MOL000228 | ADRA1B |
| MOL000449 | ADRA2A |
| MOL000449 | MAOA |
| MOL000228 | MAOB |
| MOL000173 | AR |
| MOL002714 | APOD |
| MOL000173 | BAX |
| MOL000173 | BCL2 |
| MOL002714 | AHR |
| MOL000173 | BBC3 |
| MOL000449 | ADRB1 |
| MOL000173 | ADRB2 |
| MOL000073 | DPEP1 |
| MOL000552 | KCNMA1 |
| MOL000073 | PRKACA |
| MOL000228 | PKIA |
| MOL002897 | PDE10A |
| MOL012245 | CA2 |
| MOL000173 | CASP3 |
| MOL000358 | CASP8 |
| MOL000173 | CASP9 |
| MOL002714 | CDK1 |
| MOL000173 | CDK2 |
| MOL002928 | CDK7 |
| MOL000173 | TP53 |
| MOL000173 | PDE3A |
| MOL000449 | CTRB1 |
| MOL000552 | F7 |
| MOL000552 | F10 |
| MOL000173 | CDKN1A |
| MOL002714 | CYCS |
| MOL001689 | CYP19A1 |
| MOL002928 | CYP2C9 |
| MOL000228 | DRD1 |
| MOL000173 | DPP4 |
| MOL000552 | TOP2A |
| MOL002714 | EGLN1 |
| MOL000073 | ESR1 |
| MOL000552 | ESR2 |
| MOL000173 | EIF6 |
| MOL002714 | FABP5 |
| MOL001689 | FASN |
| MOL000173 | FN1 |
| MOL002714 | FOSL1 |
| MOL002714 | FOSL2 |
| MOL000173 | CCND1 |
| MOL002714 | CCNB1 |
| MOL000173 | GABRA1 |
| MOL000358 | GABRA2 |
| MOL000358 | GABRA3 |
| MOL000358 | GABRA5 |
| MOL002909 | PYGM |
| MOL000173 | GSK3B |
| MOL000073 | HSP90AA1 |
| MOL002714 | HIF1A |
| MOL000449 | IGHG1 |
| MOL000173 | MCL1 |
| MOL002714 | IGF2 |
| MOL000173 | IL6 |
| MOL000173 | CXCL8 |
| MOL000173 | MMP1 |
| MOL000449 | LTA4H |
| MOL002714 | MMP9 |
| MOL000358 | MAP2 |
| MOL000359 | NR3C2 |
| MOL000173 | MAPK14 |
| MOL000358 | OPRM1 |
| MOL000228 | CHRM1 |
| MOL000358 | CHRM2 |
| MOL000228 | CHRM3 |
| MOL000358 | CHRM4 |
| MOL002714 | MPO |
| MOL002714 | NOX5 |
| MOL000358 | CHRNA2 |
| MOL000228 | CHRNA7 |
| MOL000552 | NOS3 |
| MOL000173 | NOS2 |
| MOL002714 | NFATC1 |
| MOL000449 | NCOA1 |
| MOL000358 | NCOA2 |
| MOL000552 | PPARD |
| MOL000173 | PPARG |
| MOL000173 | PIK3CG |
| MOL000358 | KCNH2 |
| MOL000358 | PGR |
| MOL000173 | PTGER3 |
| MOL000073 | PTGS1 |
| MOL000073 | PTGS2 |
| MOL000358 | PRKCA |
| MOL000173 | PRKCD |
| MOL000552 | F2 |
| MOL002714 | FOS |
| MOL000173 | AKT1 |
| MOL000173 | RXRA |
| MOL000173 | CHEK1 |
| MOL000358 | PON1 |
| MOL000173 | CCL2 |
| MOL000228 | SLC6A3 |
| MOL000449 | SLC6A2 |
| MOL000228 | SLC6A4 |
| MOL000173 | SCN5A |
| MOL000173 | TEP1 |
| MOL000173 | JUN |
| MOL000173 | RELA |
| MOL000358 | TGFB1 |
| MOL000173 | PRSS1 |
| MOL002714 | TDRD7 |
| MOL000173 | TNF |
| MOL001689 | FASLG |
| MOL002909 | PTPN1 |
| MOL000449 | PLAU |
| MOL000173 | KDR |
| MOL002927 | CACNA2D1 |
|  |  |
| Panax Ginseng C. A. Mey. |  |
| Mol ID | gene |
| MOL000422 | PSMD3 |
| MOL000358 | HTR2A |
| MOL000422 | ACHE |
| MOL000422 | AHSA1 |
| MOL000422 | AKR1C3 |
| MOL000449 | ALDRA |
| MOL000358 | ADRA1A |
| MOL000358 | ADRA1B |
| MOL000787 | ADRA1D |
| MOL000449 | ADRA2A |
| MOL000449 | MAOA |
| MOL000449 | MAOB |
| MOL000422 | AR |
| MOL000422 | SLPI |
| MOL000358 | BAX |
| MOL000358 | BCL2 |
| MOL000422 | ALOX5 |
| MOL000422 | AHR |
| MOL000449 | ADRB1 |
| MOL000358 | ADRB2 |
| MOL005384 | KCNMA1 |
| MOL000358 | PRKACA |
| MOL000787 | PDE4D |
| MOL005344 | CASP1 |
| MOL000358 | CASP3 |
| MOL000358 | CASP8 |
| MOL000358 | CASP9 |
| MOL000422 | CDK1 |
| MOL000358 | PDE3A |
| MOL000449 | CTRB1 |
| MOL000422 | F7 |
| MOL000787 | F10 |
| MOL000422 | CYP1A1 |
| MOL000422 | CYP1B1 |
| MOL000422 | CYP3A4 |
| MOL000358 | DRD1 |
| MOL000787 | OPRD1 |
| MOL000422 | DPP4 |
| MOL000422 | TOP2A |
| MOL005344 | MAP2K4 |
| MOL000422 | SELE |
| MOL000358 | GABRA1 |
| MOL000358 | GABRA2 |
| MOL000358 | GABRA3 |
| MOL000358 | GABRA5 |
| MOL005376 | NR3C1 |
| MOL000422 | GSTM1 |
| MOL000422 | GSTM2 |
| MOL000358 | HSP90AA1 |
| MOL000422 | HMOX1 |
| MOL000422 | HAS2 |
| MOL000449 | IGHG1 |
| MOL000422 | IKBKB |
| MOL000422 | INSR |
| MOL000422 | ICAM1 |
| MOL005344 | IFNG |
| MOL005344 | IL1B |
| MOL000422 | MMP1 |
| MOL000449 | LTA4H |
| MOL000358 | MAP2 |
| MOL000449 | NR3C2 |
| MOL000422 | MAPK8 |
| MOL000358 | OPRM1 |
| MOL000358 | CHRM1 |
| MOL000358 | CHRM2 |
| MOL000358 | CHRM3 |
| MOL000358 | CHRM4 |
| MOL000787 | CHRM5 |
| MOL000358 | CHRNA2 |
| MOL000358 | CHRNA7 |
| MOL005344 | NFKBIA |
| MOL000422 | NOS3 |
| MOL000422 | NOS2 |
| MOL000449 | NCOA1 |
| MOL000358 | NCOA2 |
| MOL000422 | NR1I2 |
| MOL000422 | NR1I3 |
| MOL000422 | PRXC1A |
| MOL000422 | PPARG |
| MOL000358 | PIK3CG |
| MOL005344 | ADCYAP1 |
| MOL000358 | KCNH2 |
| MOL000358 | PGR |
| MOL000358 | PTGS1 |
| MOL000358 | PTGS2 |
| MOL005344 | PSMG1 |
| MOL000358 | PRKCA |
| MOL000422 | F2 |
| MOL000422 | AKT1 |
| MOL000449 | RXRA |
| MOL005320 | RXRG |
| MOL000422 | PPP3CA |
| MOL000358 | PON1 |
| MOL000422 | STAT1 |
| MOL000449 | SLC6A3 |
| MOL000422 | SLC6A2 |
| MOL000358 | SLC6A4 |
| MOL000358 | SCN5A |
| MOL000422 | SLC2A4 |
| MOL000358 | JUN |
| MOL000422 | RELA |
| MOL000358 | TGFB1 |
| MOL000422 | PRSS1 |
| MOL000422 | TNF |
| MOL000422 | DIO1 |
| MOL005384 | PTPN1 |
| MOL000449 | PLAU |
| MOL000422 | VCAM1 |
| MOL000787 | KDR |
| MOL000787 | CACNA1S |
| MOL000422 | XDH |
|  |  |
| licorice |  |
| Mol ID | gene |
| MOL000098 | PSMD3 |
| MOL004328 | HMGCR |
| MOL000392 | HSD3B1 |
| MOL000392 | HSD3B2 |
| MOL004328 | ABAT |
| MOL000500 | HTR2A |
| MOL000098 | MMP2 |
| MOL000098 | HSPA5 |
| MOL000098 | ACACA |
| MOL000098 | ACHE |
| MOL000098 | AHSA1 |
| MOL004328 | ADIPOQ |
| MOL004328 | AKR1C1 |
| MOL000422 | AKR1C3 |
| MOL000098 | ALDRA |
| MOL000392 | ADRA1A |
| MOL000422 | ADRA1B |
| MOL001484 | ADRA1D |
| MOL000098 | MAOB |
| MOL000098 | AR |
| MOL000422 | SLPI |
| MOL004328 | APOB |
| MOL000098 | BAX |
| MOL000098 | BCL2 |
| MOL000098 | ALOX5 |
| MOL000098 | AHR |
| MOL004328 | GOT1 |
| MOL000098 | ABCG1 |
| MOL000392 | ATP5F1B |
| MOL000098 | BIRC5 |
| MOL000098 | BCL2L1 |
| MOL003896 | ADRB1 |
| MOL000098 | ADRB2 |
| MOL000392 | DPEP1 |
| MOL004806 | BACE1 |
| MOL000098 | CRP |
| MOL000098 | CXCL10 |
| MOL000098 | CXCL11 |
| MOL000098 | CXCL2 |
| MOL004966 | KCNMA1 |
| MOL000098 | PRKACA |
| MOL000392 | PKIA |
| MOL000497 | CA2 |
| MOL000098 | CASP3 |
| MOL000098 | CASP8 |
| MOL000098 | CASP9 |
| MOL000098 | CTSD |
| MOL000098 | CAV1 |
| MOL000098 | CD40LG |
| MOL000098 | CDK1 |
| MOL000239 | CDK2 |
| MOL000497 | CDK4 |
| MOL000098 | TP53 |
| MOL000392 | PDE3A |
| MOL000098 | CLDN4 |
| MOL000098 | F7 |
| MOL000098 | F10 |
| MOL000098 | COL3A1 |
| MOL000354 | CCNA2 |
| MOL000098 | CDKN1A |
| MOL004328 | CYP19A1 |
| MOL000098 | CYP1A1 |
| MOL000098 | CYP1B1 |
| MOL000098 | CYP3A4 |
| MOL002565 | DRD1 |
| MOL000098 | DCAF5 |
| MOL002565 | OPRD1 |
| MOL000098 | DPP4 |
| MOL000098 | TOP1 |
| MOL000098 | TOP2A |
| MOL000098 | DUOX2 |
| MOL000098 | SELE |
| MOL000098 | EGFR |
| MOL000354 | ESR1 |
| MOL000239 | ESR2 |
| MOL000098 | ELK1 |
| MOL000098 | EIF6 |
| MOL004328 | FASN |
| MOL000497 | FOSL2 |
| MOL000098 | CCND1 |
| MOL000098 | CCNB1 |
| MOL000098 | GABRA1 |
| MOL000422 | GABRA2 |
| MOL000098 | GJA1 |
| MOL000354 | GRIA2 |
| MOL004328 | GSR |
| MOL000098 | GSTM1 |
| MOL000098 | GSTM2 |
| MOL000354 | PYGM |
| MOL000354 | GSK3B |
| MOL000098 | HSF1 |
| MOL000098 | HSPB1 |
| MOL000098 | HSP90AA1 |
| MOL000098 | HMOX1 |
| MOL000098 | HK2 |
| MOL000098 | NKX3-1 |
| MOL000098 | HAS2 |
| MOL000098 | HIF1A |
| MOL001484 | IGHG1 |
| MOL000098 | CHUK |
| MOL000422 | IKBKB |
| MOL000098 | IGFBP3 |
| MOL000098 | IGF2 |
| MOL000098 | INSR |
| MOL000098 | ICAM1 |
| MOL000098 | IFNG |
| MOL000098 | IRF1 |
| MOL000098 | IL1A |
| MOL000098 | IL1B |
| MOL000098 | IL10 |
| MOL000098 | IL2 |
| MOL000392 | IL4 |
| MOL000098 | IL6 |
| MOL000098 | CXCL8 |
| MOL000098 | MMP1 |
| MOL003896 | LTA4H |
| MOL004328 | CES1 |
| MOL004328 | LDLR |
| MOL000098 | MGAM |
| MOL000098 | MMP9 |
| MOL004328 | MTTP |
| MOL000359 | NR3C2 |
| MOL000098 | MAPK1 |
| MOL002565 | MAPK10 |
| MOL000354 | MAPK14 |
| MOL004328 | MAPK3 |
| MOL000422 | MAPK8 |
| MOL001484 | OPRM1 |
| MOL004328 | ABCC1 |
| MOL000392 | CHRM1 |
| MOL000422 | CHRM2 |
| MOL001484 | CHRM3 |
| MOL000500 | CHRM4 |
| MOL002565 | CHRM5 |
| MOL000098 | MYC |
| MOL000098 | MPO |
| MOL000392 | SIRT1 |
| MOL000098 | NQO1 |
| MOL000392 | MT-ND6 |
| MOL002565 | CHRNA7 |
| MOL000098 | NCF1 |
| MOL000098 | NFKBIA |
| MOL000098 | NOS3 |
| MOL000098 | NOS3 |
| MOL000239 | NOS2 |
| MOL000098 | NFE2L2 |
| MOL000354 | NCOA1 |
| MOL000098 | NCOA2 |
| MOL000098 | NR1I2 |
| MOL000098 | NR1I3 |
| MOL000098 | ODC1 |
| MOL000098 | SPP1 |
| MOL000354 | OLR1 |
| MOL000098 | PRXC1A |
| MOL000098 | PPARA |
| MOL000098 | PPARD |
| MOL000098 | PPARG |
| MOL000098 | PTEN |
| MOL000098 | PIK3CG |
| MOL004328 | PLB1 |
| MOL000098 | SERPINE1 |
| MOL000098 | PARP1 |
| MOL000098 | KCNH2 |
| MOL000098 | PCOLCE |
| MOL000211 | PGR |
| MOL000098 | PTGER3 |
| MOL000098 | PTGS1 |
| MOL000098 | PTGS2 |
| MOL000098 | ACP3 |
| MOL000098 | RUNX1T1 |
| MOL000098 | PRKCA |
| MOL000098 | PRKCB |
| MOL000098 | F2 |
| MOL000098 | FOS |
| MOL000098 | NPEPPS |
| MOL000098 | AKT1 |
| MOL000098 | RAF1 |
| MOL000098 | RASSF1 |
| MOL000098 | RASA1 |
| MOL000098 | ERBB2 |
| MOL000098 | ERBB3 |
| MOL000098 | RB1 |
| MOL000098 | RXRA |
| MOL000098 | RUNX2 |
| MOL000239 | CHEK1 |
| MOL000098 | CHEK2 |
| MOL000422 | PPP3CA |
| MOL000098 | PON1 |
| MOL000098 | STAT1 |
| MOL000497 | STAT3 |
| MOL000098 | CCL2 |
| MOL000392 | SLC6A3 |
| MOL000422 | SLC6A2 |
| MOL000392 | SLC6A4 |
| MOL000098 | SCN5A |
| MOL000098 | SLC2A4 |
| MOL004328 | SOAT1 |
| MOL004328 | SOAT2 |
| MOL004328 | SREBF1 |
| MOL000098 | MMP3 |
| MOL000098 | SOD1 |
| MOL000098 | THBD |
| MOL000098 | PLAT |
| MOL000098 | F3 |
| MOL000098 | JUN |
| MOL000098 | E2F1 |
| MOL000098 | E2F2 |
| MOL000098 | RELA |
| MOL000098 | TGFB1 |
| MOL000098 | PRSS1 |
| MOL000098 | TNF |
| MOL000098 | DIO1 |
| MOL000354 | PTPN1 |
| MOL000098 | PLAU |
| MOL000098 | VCAM1 |
| MOL002311 | KDR |
| MOL000098 | XDH |
|  |  |
| Zingiber Officinale Roscoe |  |
| Mol ID | gene |
| MOL000358 | HTR2A |
| MOL000449 | ALDRA |
| MOL000358 | ADRA1A |
| MOL000358 | ADRA1B |
| MOL000449 | ADRA2A |
| MOL000449 | MAOA |
| MOL000449 | MAOB |
| MOL000358 | BAX |
| MOL000358 | BCL2 |
| MOL000449 | ADRB1 |
| MOL000358 | ADRB2 |
| MOL000358 | PRKACA |
| MOL000358 | CASP3 |
| MOL000358 | CASP8 |
| MOL000358 | CASP9 |
| MOL000358 | PDE3A |
| MOL000449 | CTRB1 |
| MOL006129 | F10 |
| MOL000358 | DRD1 |
| MOL006129 | ESR1 |
| MOL000358 | GABRA1 |
| MOL000358 | GABRA2 |
| MOL000358 | GABRA3 |
| MOL000358 | GABRA5 |
| MOL000358 | HSP90AA1 |
| MOL000449 | IGHG1 |
| MOL000449 | LTA4H |
| MOL000358 | MAP2 |
| MOL000449 | NR3C2 |
| MOL000358 | OPRM1 |
| MOL000358 | CHRM1 |
| MOL000358 | CHRM2 |
| MOL000358 | CHRM3 |
| MOL000358 | CHRM4 |
| MOL000358 | CHRNA2 |
| MOL000358 | CHRNA7 |
| MOL000449 | NCOA1 |
| MOL000358 | NCOA2 |
| MOL000358 | PIK3CG |
| MOL000358 | KCNH2 |
| MOL000358 | PGR |
| MOL000358 | PTGS1 |
| MOL000358 | PTGS2 |
| MOL000358 | PRKCA |
| MOL000449 | RXRA |
| MOL000358 | PON1 |
| MOL000449 | SLC6A3 |
| MOL000449 | SLC6A2 |
| MOL000358 | SLC6A4 |
| MOL000358 | SCN5A |
| MOL000358 | JUN |
| MOL000358 | TGFB1 |
| MOL000449 | PLAU |
|  |  |
| Jujubae Fructus |  |
| Mol ID | gene |
| MOL000098 | PSMD3 |
| MOL000358 | HTR2A |
| MOL000627 | HTR2C |
| MOL000098 | MMP2 |
| MOL000098 | HSPA5 |
| MOL000098 | ACACA |
| MOL000098 | ACHE |
| MOL000098 | AHSA1 |
| MOL000098 | ALDRA |
| MOL000358 | ADRA1A |
| MOL000358 | ADRA1B |
| MOL000627 | ADRA1D |
| MOL000449 | ADRA2A |
| MOL000627 | ADRA2B |
| MOL000627 | ADRA2C |
| MOL000449 | MAOA |
| MOL000098 | MAOB |
| MOL000098 | AR |
| MOL000098 | BAX |
| MOL000098 | BCL2 |
| MOL000098 | ALOX5 |
| MOL000098 | AHR |
| MOL000098 | ABCG1 |
| MOL000098 | BIRC5 |
| MOL000098 | BCL2L1 |
| MOL000449 | ADRB1 |
| MOL000098 | ADRB2 |
| MOL000096 | DPEP1 |
| MOL000098 | CRP |
| MOL000098 | CXCL10 |
| MOL000098 | CXCL11 |
| MOL000098 | CXCL2 |
| MOL000096 | PRKACA |
| MOL000787 | PDE4D |
| MOL001454 | PDE10A |
| MOL000627 | CA2 |
| MOL000098 | CASP3 |
| MOL002773 | CASP7 |
| MOL000098 | CASP8 |
| MOL000098 | CASP9 |
| MOL002773 | CTNNB1 |
| MOL000098 | CTSD |
| MOL000098 | CAV1 |
| MOL000098 | CD40LG |
| MOL000098 | CDK1 |
| MOL000098 | TP53 |
| MOL000358 | PDE3A |
| MOL000449 | CTRB1 |
| MOL000098 | CLDN4 |
| MOL000098 | F7 |
| MOL000098 | F10 |
| MOL000098 | COL3A1 |
| MOL000098 | CDKN1A |
| MOL000098 | CYP1A1 |
| MOL000098 | CYP1B1 |
| MOL012976 | CYP2B6 |
| MOL000098 | CYP3A4 |
| MOL000358 | DRD1 |
| MOL000627 | DRD5 |
| MOL007213 | DRD2 |
| MOL000627 | DRD4 |
| MOL000098 | DCAF5 |
| MOL000627 | OPRD1 |
| MOL000098 | DPP4 |
| MOL000098 | TOP1 |
| MOL000098 | TOP2A |
| MOL000098 | DUOX2 |
| MOL000098 | SELE |
| MOL000098 | EGFR |
| MOL000096 | ESR1 |
| MOL000098 | ELK1 |
| MOL000098 | EIF6 |
| MOL000096 | FASN |
| MOL000098 | CCND1 |
| MOL000098 | CCNB1 |
| MOL000098 | GABRA1 |
| MOL000358 | GABRA2 |
| MOL000358 | GABRA3 |
| MOL000358 | GABRA5 |
| MOL000098 | GJA1 |
| MOL000098 | GSTM1 |
| MOL000098 | GSTM2 |
| MOL000098 | HSF1 |
| MOL000098 | HSPB1 |
| MOL000096 | HSP90AA1 |
| MOL000098 | HMOX1 |
| MOL000098 | HK2 |
| MOL000098 | NKX3-1 |
| MOL012976 | CDC37 |
| MOL000098 | HAS2 |
| MOL000098 | HIF1A |
| MOL000449 | IGHG1 |
| MOL000098 | CHUK |
| MOL000098 | IGFBP3 |
| MOL000098 | IGF2 |
| MOL000098 | INSR |
| MOL000098 | ICAM1 |
| MOL000098 | IFNG |
| MOL000098 | IRF1 |
| MOL000098 | IL1A |
| MOL000098 | IL1B |
| MOL000098 | IL10 |
| MOL000098 | IL2 |
| MOL000098 | IL6 |
| MOL000098 | CXCL8 |
| MOL000098 | MMP1 |
| MOL000096 | KLF7 |
| MOL000449 | LTA4H |
| MOL000098 | MGAM |
| MOL000098 | MMP9 |
| MOL000358 | MAP2 |
| MOL000449 | NR3C2 |
| MOL000098 | MAPK1 |
| MOL000358 | OPRM1 |
| MOL000358 | CHRM1 |
| MOL000358 | CHRM2 |
| MOL000358 | CHRM3 |
| MOL000358 | CHRM4 |
| MOL000627 | CHRM5 |
| MOL000098 | MYC |
| MOL000098 | MPO |
| MOL000098 | NQO1 |
| MOL000358 | CHRNA2 |
| MOL000358 | CHRNA7 |
| MOL000098 | NCF1 |
| MOL000098 | NFKBIA |
| MOL000098 | NOS3 |
| MOL000098 | NOS3 |
| MOL001454 | NOS2 |
| MOL000098 | NFE2L2 |
| MOL000449 | NCOA1 |
| MOL000096 | NCOA2 |
| MOL000098 | NR1I2 |
| MOL000098 | NR1I3 |
| MOL000098 | ODC1 |
| MOL000098 | SPP1 |
| MOL000098 | PRXC1A |
| MOL000098 | PPARA |
| MOL000098 | PPARD |
| MOL000096 | PPARG |
| MOL000098 | PTEN |
| MOL000098 | PIK3CG |
| MOL000098 | SERPINE1 |
| MOL000098 | PARP1 |
| MOL000098 | KCNH2 |
| MOL000098 | PCOLCE |
| MOL000211 | PGR |
| MOL000098 | PTGER3 |
| MOL000096 | PTGS1 |
| MOL000096 | PTGS2 |
| MOL000098 | ACP3 |
| MOL000098 | RUNX1T1 |
| MOL000098 | PRKCA |
| MOL000098 | PRKCB |
| MOL000098 | F2 |
| MOL000098 | FOS |
| MOL000098 | NPEPPS |
| MOL000098 | AKT1 |
| MOL000098 | RAF1 |
| MOL000098 | RASSF1 |
| MOL000098 | RASA1 |
| MOL000098 | ERBB2 |
| MOL000098 | ERBB3 |
| MOL000098 | RB1 |
| MOL000098 | RXRA |
| MOL000098 | RUNX2 |
| MOL000098 | CHEK2 |
| MOL000098 | PON1 |
| MOL000098 | STAT1 |
| MOL000098 | CCL2 |
| MOL000449 | SLC6A3 |
| MOL000449 | SLC6A2 |
| MOL000358 | SLC6A4 |
| MOL000098 | SCN5A |
| MOL000098 | SLC2A4 |
| MOL000098 | MMP3 |
| MOL000098 | SOD1 |
| MOL000098 | THBD |
| MOL000098 | PLAT |
| MOL000098 | F3 |
| MOL000098 | JUN |
| MOL000098 | E2F1 |
| MOL000098 | E2F2 |
| MOL000098 | RELA |
| MOL000098 | TGFB1 |
| MOL000098 | PRSS1 |
| MOL000098 | TNF |
| MOL000098 | DIO1 |
| MOL000098 | PLAU |
| MOL000098 | VCAM1 |
| MOL000787 | KDR |
| MOL000787 | CACNA1S |
| MOL000098 | XDH |
